# Screening of 5- and 6-Substituted Amiloride Libraries Identifies Dual-uPA/NHE1 Active and Single Target-Selective Inhibitors

**DOI:** 10.3390/ijms22062999

**Published:** 2021-03-15

**Authors:** Benjamin J. Buckley, Ashna Kumar, Ashraf Aboelela, Richard S. Bujaroski, Xiuju Li, Hiwa Majed, Larry Fliegel, Marie Ranson, Michael J. Kelso

**Affiliations:** 1 Illawarra Health and Medical Research Institute, Wollongong, NSW 2522, Australia; aak472@uowmail.edu.au (A.K.); ashrafam@uow.edu.au (A.A.); rb834@uowmail.edu.au (R.S.B.); hham986@uowmail.edu.au (H.M.); mranson@uow.edu.au (M.R.); 2 School of Chemistry and Molecular Bioscience, University of Wollongong, Wollongong, NSW 2522, Australia; 3 Molecular Horizons, University of Wollongong, Wollongong, NSW 2522, Australia; 4 CONCERT-Translational Cancer Research Centre, Sydney, NSW 2750, Australia; 5 Department of Biochemistry, University of Alberta, Edmonton, AB T6G 2H7, Canada; xjli@ualberta.ca (X.L.); lfliegel@ualberta.ca (L.F.)

**Keywords:** sodium-hydrogen exchanger isoform-1, NHE1, amiloride, urokinase-type plasminogen activator, uPA, cancer, metastasis

## Abstract

The K^+^-sparing diuretic amiloride shows off-target anti-cancer effects in multiple rodent models. These effects arise from the inhibition of two distinct cancer targets: the trypsin-like serine protease urokinase-type plasminogen activator (uPA), a cell-surface mediator of matrix degradation and tumor cell invasiveness, and the sodium-hydrogen exchanger isoform-1 (NHE1), a central regulator of transmembrane *p*H that supports carcinogenic progression. In this study, we co-screened our library of 5- and 6-substituted amilorides against these two targets, aiming to identify single-target selective and dual-targeting inhibitors for use as complementary pharmacological probes. Closely related analogs substituted at the 6-position with pyrimidines were identified as dual-targeting (pyrimidine **24** uPA IC_50_ = 175 nM, NHE1 IC_50_ = 266 nM, uPA selectivity ratio = 1.5) and uPA-selective (methoxypyrimidine **26** uPA IC_50_ = 86 nM, NHE1 IC_50_ = 12,290 nM, uPA selectivity ratio = 143) inhibitors, while high NHE1 potency and selectivity was seen with 5-morpholino (**29** NHE1 IC_50_ = 129 nM, uPA IC_50_ = 10,949 nM; NHE1 selectivity ratio = 85) and 5-(1,4-oxazepine) (**30** NHE1 IC_50_ = 85 nM, uPA IC_50_ = 5715 nM; NHE1 selectivity ratio = 67) analogs. Together, these amilorides comprise a new toolkit of chemotype-matched, non-cytotoxic probes for dissecting the pharmacological effects of selective uPA and NHE1 inhibition versus dual-uPA/NHE1 inhibition.

## 1. Introduction

Regulation of transmembrane *p*H gradients is an essential requirement for cellular homeostasis and healthy function in virtually all cell types. Mammalian cells typically maintain a shallow *p*H gradient through plasma membrane transporters and ion channels such that the cytosol is slightly more acidic than the extracellular fluid [1]. Reversal of transmembrane *p*H gradients is a recognised hallmark of neoplastic transformation and is observed in virtually all cancer cells [2]. The sodium-hydrogen exchanger isoform-1 (NHE1) is a ubiquitously expressed regulator of transmembrane *p*H that functions in the isoelectronic exchange of intracellular H^+^ for extracellular Na^+^ with 1:1 stoichiometry [3]. Under conditions of intracellular acidification, NHE1 is allosterically activated by cytosolic H^+^, resulting in net acid extrusion coupled to the matched influx of Na^+^ down a transmembrane gradient [4]. In multiple cancers, upregulation of acidic metabolism (i.e., the Warburg Effect) [5] promotes activation of NHE1, leading to acidification of the extracellular tumor microenvironment and alkalinisation of cytosolic *p*H [6]. These transmembrane *p*H perturbations confer a survival advantage to transformed cells, ultimately promoting carcinogenic progression [7]. Accordingly, the development of new drugs targeting NHE1 and cancer-specific *p*H dysregulation is an area of significant interest [8,9,10,11].

A second anti-cancer target associated with metastatic progression is the trypsin-like serine protease (TLSP) urokinase-type plasminogen activator (uPA). uPA specifically activates plasminogen to the broad-spectrum TLSP plasmin, a ‘master-switch’ enzyme that directly degrades components of the extracellular matrix (ECM) and activates a cascade of downstream regulators, including pro-matrix metalloproteinases and the release of latent growth factors from the ECM [12]. The primary function of uPA is to control plasminogen activation at the surface of migratory cells, a process that is coordinated by association with its cognate glycosylphosphatidylinositol-linked receptor, urokinase plasminogen activator receptor (uPAR), and plasminogen co-localised at the cell surface [13]. uPA and uPAR expression is increased in multiple aggressive solid tumors, leading to proteolysis and remodelling of the tumor microenvironment that ultimately promotes metastatic spread [14]. Thus, small molecule inhibitors targeting the proteolytic activity of uPA hold considerable promise as anti-metastasis drugs [15].

Amiloride (AML) is a K^+^-sparing diuretic that has been used since the late 1960s to manage hypertension and congestive heart failure [16,17]. Amiloride exerts its clinical effects through inhibition of renal epithelial sodium channels (ENaC), [18,19] with on-target ENaC-mediated hyperkalemia the only notable safety risk at clinically relevant doses [20,21]. Aside from its clinical use, amiloride shows robust anti-cancer effects in multiple rodent models at supra-clinical doses [22,23]. These effects appear to arise from the drug’s moderate inhibitory activity (low µM) against NHE1 or uPA, or possibly dual activity at both targets [24]. On the basis of this premise, we initiated a medicinal chemistry program aiming to identify amiloride analogs with increased potency against these targets for possible use as new cancer drugs. Our initial efforts to increase uPA potency by adding substituted amines to position 5 on the pyrazine core were largely unsuccessful, producing only modest improvements (~2-fold) relative to amiloride [25]. More recent work with matched series of 6-substituted 5-(*N*,*N*-hexamethylene)amiloride (HMA) [26] and amiloride (i.e., 5-NH_2_) [27] analogs targeting the uPA S1β subsite resulted in uPA potency gains exceeding 100-fold. Lead compounds from these series showed low nM uPA potency, high selectivity for uPA across the serine hydrolase superfamily, no ENaC activity, and in vivo anti-metastatic effects in xenografted mouse models of lung and pancreatic cancer [26,27]. Activity against NHE1 was not examined in these studies.

Structure–activity relationships (SAR) of amiloride analogs against NHE1 have been reported for most positions around the pyrazine core [28]. The 5-NH_2_ group was found to tolerate a wide range of alkyl and alkylaryl substituents, with higher potency generally seen for dialkyl amines over monoalkyl-substituted compounds [29,30]. For example, HMA and 5-(*N*-ethyl-*N*-isopropyl)amiloride (EIPA) are active inhibitors [28,31,32]. The 2-position acylguanidine is essential for activity and alkyl or aryl substitution of the guanidino terminal nitrogen is not tolerated [30]. This contrasts with trends seen in other trimeric ENaC/Degenerin superfamily members, e.g., ENaC [31,33] and acid-sensing ion channels (ASICs) [34], where guanidine substitution generally improves activity. Reports on amilorides carrying variations at the 6-position have been limited to 6-H or 6-halo analogs [30], where moderate improvements (up to 5–fold) are seen with increasing halogen size.

To better understand the anti-cancer mechanisms of amilorides, we set out to identify complementary pharmacological probes that could be used in future studies to unravel the relative effects of uPA versus NHE1 versus dual-inhibition. In pursuit of this, we co-screened our 5- and 6-substituted amiloride libraries for NHE1 and uPA activity and herein report the discovery of non-cytotoxic, dual-uPA/NHE1 active, and single target-selective amilorides.

## 2. Results

### 2.1. uPA Activity Screening

**Figure 1:**
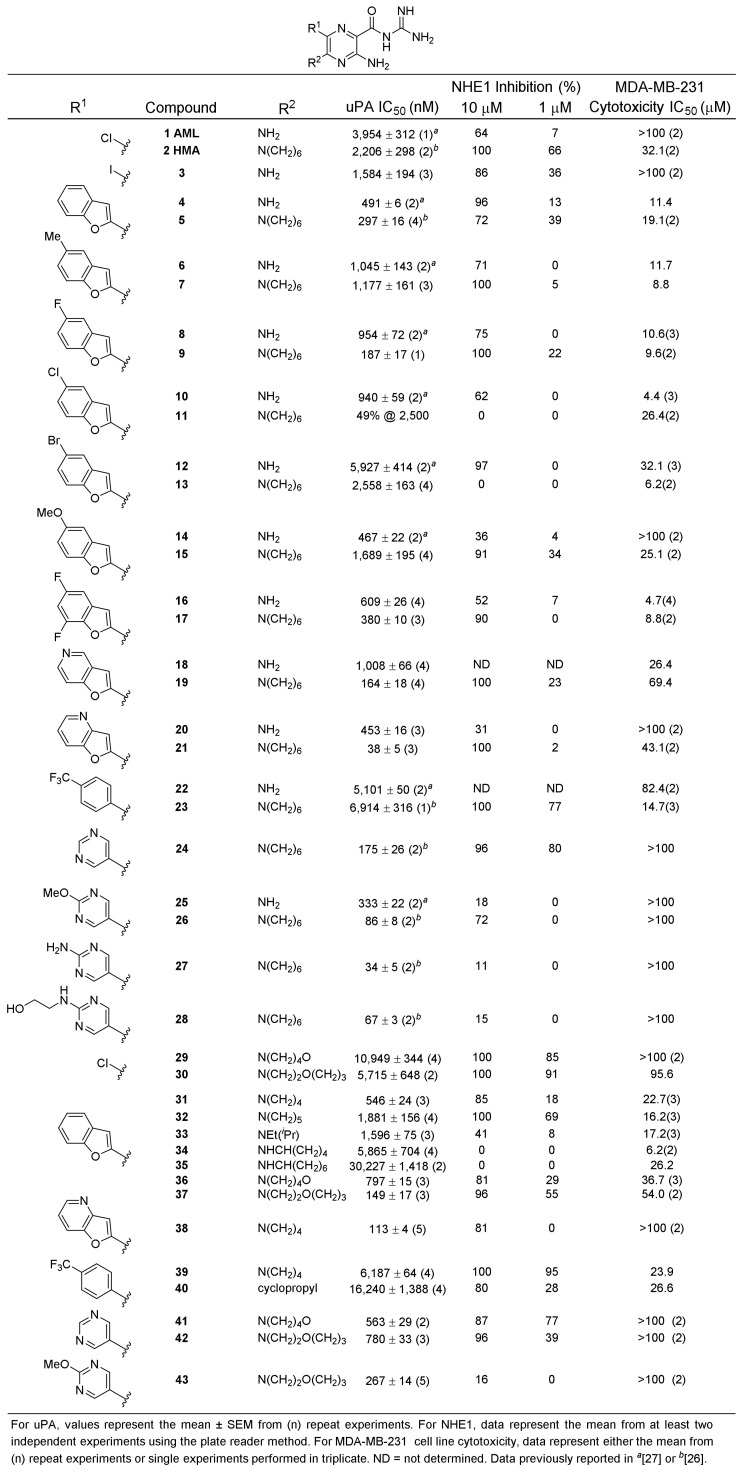
Urokinase-type plasminogen activator (uPA) and sodium-hydrogen exchanger isoform-1 (NHE1) inhibition and cytotoxicity data for 5- and 6-substituted amilorides. The trend was also seen with other substituted pyrimidines, with the amino **27** and ethanolamine **28** substituted HMA analogs and methoxypyrimidine amiloride **25** showing only 11–18% inhibition at 10 µM. Collectively, the uPA and preliminary NHE1 screening data indicate that the substituted pyrimidines are potentially compounds with selectivity for uPA. Additionally, the data indicate that the parent unsubstituted pyrimidine **24** is a possible dual-targeting uPA/NHE1 inhibitor, and that the 6-Cl-5-morpholino **29**, 1,4-oxazepine **30**, and the 6-(4-CF_3_-phenyl) pyrrolidine **39** analogs are potential NHE1-selective inhibitors.

Our libraries (containing reported and several previously undisclosed analogs) were screened for uPA activity using the reported fluorescence-based assay (Figure 1) [26]. The goal was to identify structurally related compounds showing a range of inhibitory potencies which, when co-screened against NHE1, would identify single-target (i.e., uPA or NHE1) selective and dual-uPA/NHE1 active inhibitors. The libraries contained a variety of 6-(het)aryl-substituted AML and HMA matched pairs, along with distinct sub-series, wherein additional changes were made either on the 6-substituent itself or at position 5. Inhibition was generally higher for 6-(2-benzofuranyl) HMA analogs (**5**, **9**, **13**, **17**) relative to their corresponding amiloride (5-NH_2_) derivatives (**4**, **8**, **12**, **16**; Figure 1). A notable exception was the 5-methoxy substituted **14**, which showed >3-fold higher activity than its HMA congener **15**. Substitution at the 5-position of the benzofuran ring was not favoured in either series except for 5-fluoro HMA analog **9**, which showed a modest improvement relative to the unsubstituted parent **5**. 5-Furopyridine **19** (uPA IC_50_ = 164 nM) and 4-furopyridine **21** (uPA IC_50_ = 38 nM) HMA analogs each showed significantly higher potency than the corresponding amilorides, **18** (uPA IC_50_ = 1,008 nM) and **20** (uPA IC_50_ = 453 nM). Varying the size of the 5-alkylamino group within the 6-(2-benzofuranyl) series produced modest decreases in activity (**31** and **32**). Opening the ring (ethyl isopropyl derivative **33**) decreased activity >5-fold. Activity decreased further for cyclopentylamine **34**, 5-cycloheptylamine **35** derivatives, the 5-morpholino **29,** and 5-(1,4-oxazepine) **30** substituted amilorides. Similar decreases were not seen for 5-morpholino **36** and 5-(1,4-oxazepine) **37** derivatives containing a benzofuran at the 6-position, nor for pyrimidine derivatives **41**–**43**. The previously reported pyrimidine **24** (uPA IC_50_ = 175 nM) and methoxypyrimidine **26** (uPA IC_50_ = 86 nM) derivatives were notable for their strong uPA activity and contrasting effects on NHE1 (see below).

### 2.2. NHE1 Activity Screening

To perform NHE1 screening with increased throughput, we adapted the conventional cuvette-based NHE1 activity assay to a simple new 96-well plate format using MDA-MB-231 cells that were compatible with common laboratory fluorescence plate readers (see Section 4 for details) [35,36]. The new format allowed screening of up to 12 compounds alongside vehicle and the 100% inhibition control (BI-9627, 1 µM [36], Figure S1) on a single assay plate. AML, HMA, and compounds **3**–**43** were initially screened for NHE1 % inhibition at two concentrations: 1 µM and 10 µM (Figure 1). In accordance with the literature, [30] HMA showed stronger inhibition than AML, completely blocking activity at 10 µM. Replacing the 6-Cl group of amiloride with iodine **3** was found to increase activity, also as reported [30]. Introduction of oxygen onto the 5-azepane ring of HMA (i.e., 1,4-oxazepine **30**) increased activity (91% inhibition at 1 µM) and a reduced ring size (1,4-morpholino **29**) was tolerated (85% inhibition at 1 µM). Substituting the 6-Cl group of AML with a 2-benzofuran **4** increased activity, while a small drop in activity was seen with the corresponding 5-azepane-substituted **5** relative to HMA. Improvements were generally not seen with substituted benzofuran or furopyridine analogs from either series (**6**–**21**). Similarly, improved activity was not seen for a range of 6-(2-benzofuran) analogs containing different substituents at position 5 (**31**–**37**). In contrast, strong inhibition was observed for 5-substituted 6-(4-CF_3_-phenyl) derivatives, with the 5-pyrrolidine analog **39** showing the highest activity of all analogs tested (95% at 1 µM). Good inhibition was seen with the pyrimidine-substituted analog **24** (80% inhibition at 1 µM), suggesting it as a possible dual-uPA/NHE1 active candidate. The pyrimidine series proved highly sensitive to substitution, with the methoxy-substituted **26** showing no activity at 1 µM. 

### 2.3. Mammalian Cell Cytotoxicity Screening

For selective uPA, NHE1, and dual-targeting inhibitors to be useful pharmacological probes for future cell-based studies, the compounds need to be non-cytotoxic. Accordingly, AML, HMA, and compounds **3**–**43** were screened for cytotoxicity in MDA-MB-231 cells [26] (Figure 1). Cytotoxicity generally aligned with the identity and hydrophobicity of the 6-substituent, where 6-Cl and 6-pyrimidine substituted analogs were less toxic than the more hydrophobic 6-benzofuran and 6-(4-CF_3_-phenyl) variants. The most promising dual-uPA/NHE1 active inhibitor **24**, uPA selective **26**, and NHE-selective **29**/**30** candidates all showed low toxicity, with IC_50_ values above or just below 100 µM.

### 2.4. NHE1 IC_50_ Measurements

**Figure 2:**
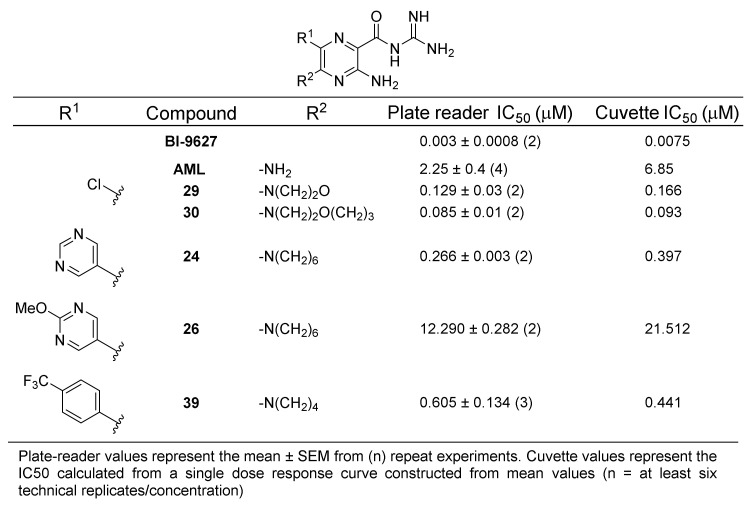
Comparative NHE1 inhibition data (IC_50_) obtained using plate-reader and cuvette fluorescence assays.

**Figure 3:**
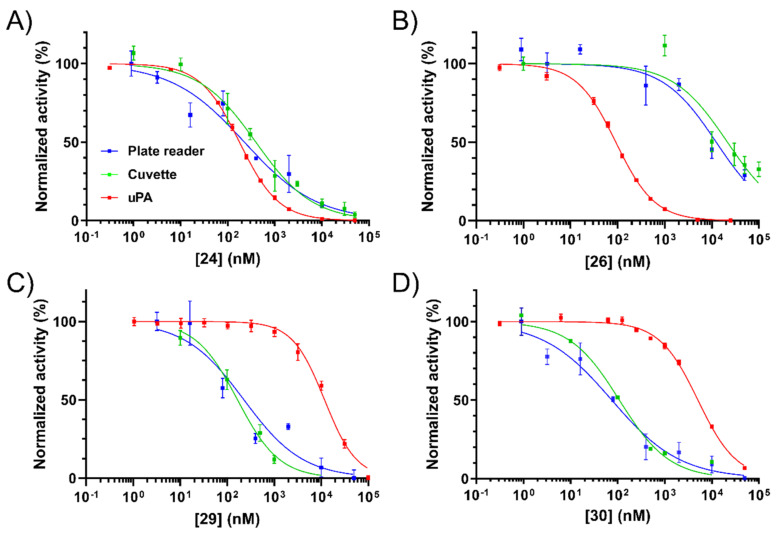
Representative NHE1 and uPA inhibition curves for: (**A**) **24**, (**B**) **26**, (**C**) **29**, and (**D**) **30**. Blue = NHE1 inhibition determined using the plate-reader assay. Green = NHE1 inhibition determined using the cuvette assay. Red = inhibition of human uPA activity. Data points = mean ± SEM; blue and red (*n* = 3), and green (*n* = at least six technical replicates/concentration).

With a shortlist of non-toxic candidate inhibitors identified, we then sought to confirm and more accurately characterise NHE1 activity by measuring IC_50_ values. Control compounds amiloride and the potent NHE1 inhibitor BI-9627 [36] were included for comparison. Minor modifications to the plate reader assay allowed accurate measurement of the concentration–response curves. To validate the new method, reference curves and IC_50_ values were obtained in parallel using the standard cuvette-based assay [35]. IC_50_ data from the two assays are presented in Figure 2. NHE1 inhibition curves for the key compounds **24**, **26**, **29**, and **30** are presented alongside their uPA inhibition curves in Figure 3.

IC_50_ values from the plate assay were consistent with the cuvette-based measurements, with the rank order of compound potencies identical in each case. The positive control NHE1 inhibitor BI-9627 showed very high potency as expected, with IC_50_ values from both assays comparing well with the literature (6 nM) [36]. Similarly consistent findings were seen with AML (3 µM) [37]. Compared to the cuvette assay, the plate reader method generally returned lower IC_50_ values, but the differences were ~2-fold or less. From these experiments, 5-substituted amilorides **29** and **30** were confirmed as potent NHE1 inhibitors showing high selectivity over uPA (85- and 67-fold, respectively). Earlier observations from the preliminary NHE1 screen showing high sensitivity of 6-pyrimidine analogs to substitution were recapitulated in the IC_50_ measurements, where the methoxy-substituted pyrimidine **26** showed an ~46-fold drop in potency relative to unsubstituted **24**. Thus, compounds **24** (uPA selectivity ratio = 1.5) and **26** (uPA selectivity ratio = 143) were confirmed as dual-uPA/NHE1 active and uPA selective inhibitors, respectively. The very strong inhibition seen with 6-(4-CF_3_-phenyl) compound **39** in the NHE1 screening assay was not seen in the dose-response experiments. This lower-than-expected activity, coupled with higher cytotoxicity, excluded it from further consideration.

### 2.5. Inhibition of uPA Activity at the Cell Surface

**Figure 4:**
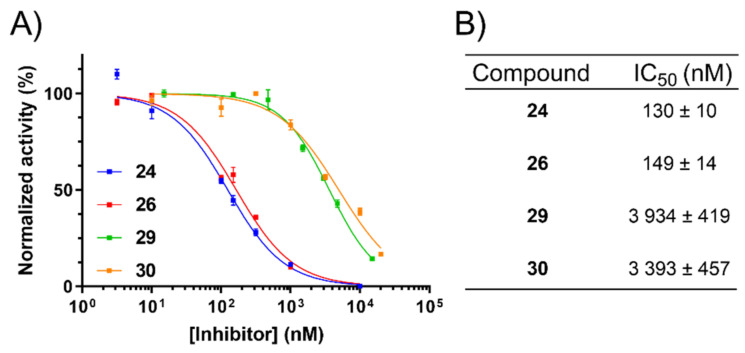
Inhibition of MDA-MB-231 cell-surface uPA activity. (**A**) Dose-response curves for **24**, **26**, **29**, and **30**. Data represent the mean ± SEM (*n* = three technical replicates/concentration). (**B**) Average IC_50_ values ± SEM from four independent assays.

Having identified non-cytotoxic compounds with the desired target selectivity profiles, we then sought to confirm their uPA inhibitory activities in a more physiologically relevant, whole-cell assay. To this end, the fluorogenic biochemical assay was modified to allow measurement of cell-surface uPA activity in MDA-MB-231 cells, which are known to express uPAR [38,39]. To maximise enzymatic activity, the cells were pre-incubated with active high molecular weight (HMW) uPA to saturate unoccupied uPAR present at the cell surface. The data obtained compared very well to the purified enzyme assay with IC_50_ values differing across formats by less than 2–3-fold for all four compounds (Figure 4).

## 3. Discussion

In this study, we identified 6-substituted amiloride and HMA analogs showing dual- and single-target selective activity against uPA and NHE1. Specifically, pyrimidine-substituted HMA analog **24** showed strong activity (IC_50_ < 300 nM) at both targets in biochemical and cell assays, as well as minimal effects on cell viability. While a number of other analogs showed slightly lower dual-activity (IC_50_ <600 nM), suggesting that NHE1 was generally tolerant of 6-(het)aryl substitutions, a remarkable degree of uPA selectivity was observed with the methoxypyrimidine **26**. The 6-(4-CF_3_-phenyl) **39** initially appeared as the most selective NHE1 inhibitor. However, the compound showed significant cytotoxicity. The superior potency and low cytotoxicity of 6-Cl 5-morpholino **29** and 5-(1,4-oxazepine) **30** marked these analogs as excellent NHE1-selective inhibitors. These findings shed new light on our previous results demonstrating the anti-metastatic properties of **26** in an orthotopic xenograft model of pancreatic ductal adenocaricinoma [26], an aggressive cancer known to overexpress uPA/uPAR [40]. The high uPA selectivity of **26** found here confirms that its anti-metastatic properties are mediated by inhibition of uPA with little or no contribution from effects on NHE1. Furthermore, the low cytotoxicity of **26** indicates that the observed efficacy was not due to direct killing of xenografted cancer cells.

Amilorides hold a singular place in the history of cell physiology, providing a set of structurally-related analogs that can inhibit several different biological targets [28]. However, numerous studies have attributed pharmacological effects to a specific target of interest following treatment with amiloride or an analog without consideration of possible off-target effects [41,42,43]. In the cancer field alone, there are a several examples whereby effects have been ascribed to inhibition of either uPA [44,45,46] or NHE1 [47,48,49] without controlling for possible effects from the other target. The situation is further confounded in studies that use amiloride as a “specific inhibitor” due to possible effects from ENaC. In recent years, ENaC has been shown to play a functional role in tissues well beyond its clinically relevant expression in the kidney [50].

The tool compounds identified herein provide an unprecedented degree of selectivity among amilorides for these two targets, which have historically been studied using non-selective analogs [51]. We previously showed that 6-(het)aryl analogs like **24** and **26** have no ENaC activity in vitro and no K^+^-sparing or diuretic effects in vivo. Additionally, the known propensity of 5-substitution to remove ENaC activity from amilorides indicates that NHE1-selective compounds **29** and **30** would similarly lack these activities [17]. The combination of these characteristics, along with low eukaryotic cell cytotoxicity, supports the use of these four amilorides as chemotype-matched, complementary pharmacological tools for cell-based studies investigating uPA and NHE1-mediated processes. In particular, the compounds represent a useful new chemical toolkit for studying the effects of singular NHE1 or uPA inhibition versus dual-uPA/NHE1 inhibition on cancer cell phenotypes.

## 4. Materials and Methods

### 4.1. uPA Inhibition Assays

Detailed methods are described in [26]. Briefly, serial dilutions of compounds were added to the wells of a black Greiner CELLSTAR^®^ 96-well plate on ice (catalog #655079 Greiner Bio-One GmBH, Kremsmünster, Austria) containing urokinase from human kidney cells (Cat # U4010, Sigma-Aldrich, St. Louis, MI, USA) and urokinase fluorescent substrate III (Z-Gly-Gly-Arg-AMC, Calbiochem Cat # 672159, Merck Millipore, Massachusetts, USA) to a final volume of 200 μL/well, final enzyme concentration 0.75 nM, and final substrate concentration 250 μM in assay buffer: 20 mM HEPES *p*H 7.4, 100 mM NaCl, 0.5 mM EDTA, 0.01% *v*/*v* Tween-20. Reaction progress was monitored immediately following addition of the enzyme using a POLARstar OMEGA (BMG Labtech GmbH, Offenburg, Germany) fluorescence plate reader with the parameters summarised in Table S1. Changes in fluorescence over a 10–15-minute period occurring over linear portions of the reaction progress curves were used to determine IC_50_ values from blank-corrected, Log-10 transformed data graphed using GraphPad PRISM v8.0 software (GraphPad Software, San Diego, USA).

### 4.2. Cell Culture Conditions

MDA-MB-231 human breast adenocarcinoma cells were serially cultured from American Type Culture Collection (ATCC)-certified stocks in DMEM/Hi-Glucose supplemented with 10% *v*/*v* heat-inactivated foetal bovine serum (FBS) (Bovogen Biologicals, Melbourne, Australia) and incubated at 37 °C, 95% humidity, and 5% *v*/*v* CO_2_ in a Heracell 150i CO_2_ incubator (Thermo Fisher Scientific, Sydney, Australia). Cells were washed with pre-warmed Ca^2+^ and Mg^2+^-free phosphate-buffered saline (PBS) and enzymatically dissociated using Trypsin/0.05% EDTA solution (Gibco, Sydney, Australia). Cells were subcultured every 3–4 days. The maximum passage number for cell lines in all experiments was 20. Cells were routinely monitored for mycoplasma contamination and validated via short tandem repeat genomic profiling.

### 4.3. Cell Viability (Cytotoxicity) Assays

MDA-MB-231 cells were seeded at a density of 7500 cells per well (final volume 90 μL) into Greiner CELLSTAR 96-well plates (Greiner Bio-One, 655180) and incubated for ~24 h. On the day of treatment, respective compounds were serially diluted using a semi-logarithmic dilution series from 20 mM stocks in neat DMSO into DMEM-high glucose media (10% *v*/*v* FBS) in a separate 96-well plate and under sterile conditions. Thereafter, 10 μL of the diluted compounds at their respective concentrations (*n* = 4) were added to the cells. DMSO was present in all wells at a final concentration of 0.25% *v*/*v*. Vehicle media blanks or drug blanks were also included to correct for inherent colour of the compounds and phenol red-containing media. Following 48 h treatment, 20 μL CellTitre 96^®^ Aqueous One Solution Cell Proliferation Assay (Promega, Madison, USA) was added to each well, plates were incubated for 2 h, and absorbance was measured at 490 nm using a SpectraMax Plus 384-well plate reader (Molecular Devices LLC, San Jose, USA) and Softmax PRO v7.0 software. Blank-corrected data were analysed and graphed using GraphPad PRISM v8.0 software (GraphPad Software, San Diego, CA USA).

### 4.4. Plate-Based NHE1 Inhibition Assays

MDA-MB-231 cells were seeded into Greiner CELLSTAR black 96-well plates (Greiner Bio-one, 655079) at density 15,000 cells/well in a volume of 150 µL media and cultured to confluence over 72 h. Culture media were removed and replaced with 200 µL 0.2% FBS-containing media with incubation for 3–4 h to stimulate NHE1 activity [52], followed by incubation for 30 min in the presence of 5 µM 2′,7′-bis(carboxyethyl)-5(6)-carboxyfluorescein-acetoxymethyl ester (BCECF-AM, cat #ab143463, Abcam, Cambridge, United Kingdom). Cells were then washed with 100 µL/well Na^+^-free acid-load buffer: 10 mM NH_4_Cl, 1.8 mM CaCl_2_, 90 mM *N*-methyl-D-glucamine (from 1 M stock titrated to *p*H 7.4 with HCl), 5 mM glucose, 15 mM HEPES, 5 mM KCl, 1 mM MgCl_2_ (*p*H 7.5, adjusted with KOH), and incubated at 37 °C for a further 30 min. Acid-load buffer was removed and replaced with Na^+^-containing assay buffer: 100 mM NaCl, 1.8 mM CaCl_2_, 5 mM glucose, 15 mM HEPES, 5 mM KCl, 1 mM MgCl_2_ containing serial dilution of each compound or DMSO vehicle, and changes in fluorescence were read immediately using a BMG Labtech POLARstar OMEGA (BMG Labtech GmbH, Offenburg, Germany) plate reader according to the parameters in Table S1. Compounds were tested at *n* = 3 technical replicates per concentration per plate. BCECF-AM-free cells were treated with matched concentrations of each compound to correct for intrinsic fluorescence. DMSO was present in all wells at a final concentration of 1% *v*/*v*. Inhibition of NHE1-mediated *p*H_i_ recovery was determined by ratiometric calculation of the change in blank-corrected *p*H-sensitive fluorescence divided by the isobestic fluorescence for each well over time. Percentage inhibition was determined by normalisation to vehicle (100% activity) and 1 µM BI-9627 [36] (0% activity) controls present on each plate. IC_50_ values were determined through fitting of Log-10 transformed data using the “log(inhibitor) versus normalised response–variable slope” non-linear function in GraphPad PRISM v8.0 software package (GraphPad Software, San Diego, CA, USA).

### 4.5. Cuvette-Based NHE1 Inhibition Assays

Performed as described in [53]. Briefly, MDA-MB-231 cells were grown to ~80–90% confluence on glass coverslips. Then, 3 µM BCECF were loaded into cells in 400 µL serum-free medium and the fluorescence was measured using a PTI Deltascan spectrofluorometer (Horiba Ltd., Kyoto, Japan). The effect of inhibitors on NHE activity was determined using the double ammonium chloride pulse protocol [54] using Na^+^ acid-load buffer: 135 mM *N*-methyl-D-glucamine, 50 mM NH_4_Cl, 5 mM KCl, 1.8 mM CaCl_2_, 1 mM MgCl_2_, 5.5 mM glucose, and 10 mM HEPES, *p*H = 7.4, 37 °C, 3 min exposure. Δ*p*H/s during the first 20 s of recovery in Na^+^-containing medium: 135 mM NaCl, 5 mM KCl, 1.8 mM CaCl_2_, 1 mM MgCl_2_, 5.5 mM glucose, and 10 mM HEPES, *p*H 7.4, 37 °C was measured. A calibration curve of *p*Hi fluorescence was performed for every sample using nigericin [55]. Results are the mean ± SEM of at least 4 technical replicates at each concentration of drug. IC_50_ values were determined through fitting of Log-10 transformed data using the “log(inhibitor) versus normalised response–variable slope” non-linear function in GraphPad PRISM v8.0 software package (GraphPad Software, San Diego, USA).

### 4.6. Cell-Surface uPA Activity Assays

MDA-MB-231 cells were seeded into Greiner CELLSTAR black 96-well plates (Greiner Bio-one, cat #655079) at density 10,000 cells/well in a volume of 100 µL media and cultured for 48 h. On the day of the experiment, cell culture media were removed via shakeout, followed by washing with 100 µL 1 × Dulbecco’s PBS/1 mM CaCl_2_/1 mM MgCl_2_/0.1% protease-free bovine serum albumin/*p*H 7.4 wash buffer. Cells were then incubated in the presence of 25 nM HMW human active uPA (Molecular Innovations, San Diego, CA, USA, SKU: HTC-UPA) in wash buffer for 30 min at RT. Unbound HMW uPA was removed via washing twice with 100 µL wash buffer. Serial dilutions of compounds in 1× PBS at 2× the desired final assay concentration were added to the cells via multichannel pipette followed by 5 min incubation at RT. The reaction was triggered by the addition of 100 µL of 1 mM Urokinase fluorescent substrate III in 1xDulbecco’s PBS as above. All compound dilutions and control solutions were present at a final DMSO concentration of 0.25 % *v*/*v*. Plates were read immediately following the addition of substrate using a POLARstar OMEGA fluorescence plate reader (BMG Labtech GmbH, Offenburg, Germany) according to the parameters in Table S1. Changes in fluorescence over a 10–15-minute period occurring over a linear portion of the reaction progress curve were used to determine IC_50_ values from blank-corrected, log-transformed data as described above.

## Data Availability

Not applicable.

## Supplementary Materials

The following are available online at https://www.mdpi.com/1422-0067/22/6/2999/s1. Figure S1. Structure of BI-9627 and B) NHE1 and uPA inhibition curves for BI-9627. Table S1. POLARstar OMEGA settings used for plate reader uPA and NHE1 inhibition assays.

## Abbreviations

AML	Amiloride
ASIC	Acid-sensing ion channel
BCECF-AM	2′,7′-bis(carboxyethyl)-5(6)-carboxyfluorescein-acetoxymethyl ester
EIPA	5-(*N*-ethyl-*N*-isopropyl)amiloride
ENaC	Epithelial sodium channel
HMA	5-(*N*,*N*-hexamethylene)amiloride
NHE1	Na^+^/H^+^ exchanger isoform-1
SAR	Structure–activity relationship
TLSP	Trypsin-like serine protease
uPA	urokinase plasminogen activator
uPAR	urokinase plasminogen activator receptor
