# Supplementary material for: Screening of 5- and 6-Substituted Amiloride Libraries Identifies Dual-uPA/NHE1 Active and Single Target-Selective Inhibitors
Source: Int J Mol Sci. 2021 Mar 15;22(6):2999. doi: 10.3390/ijms22062999 (PMC8000185; doi:10.3390/ijms22062999)
Supplement: Supplementary file 1 [file ijms-22-02999-s001.pdf]

Supplementary Material

Screening of 5- and 6-substituted amiloride libraries identifies dual-uPA/NHE1 active and single target-selective inhibitors

Benjamin J. Buckley[1,2,3,4], Ashna Kumar[1,2,3], Ashraf Aboelela[1,2], Richard S. Bujaroski[1,2,3], Xiuju Li[5], Hiwa Majed[1,2], Larry Fliegel[5], Marie Ranson[1,2,3,4] and Michael J. Kelso[1,2,3*]

[1] Illawarra Health and Medical Research Institute, Wollongong, NSW 2522, Australia.
[2] School of Chemistry and Molecular Bioscience, University of Wollongong, NSW 2522, Australia.
[3] Molecular Horizons, University of Wollongong, NSW 2522, Australia.
[4] CONCERT-Translational Cancer Research Centre, NSW 2750, Australia.
[5] Department of Biochemistry, University of Alberta, Edmonton, Alberta, T6G 2H7, Canada.

*Correspondence authors: bbuckley@uow.edu.au, mkelso@uow.edu.au; Tel.: +61-2-4221-5085

Contents

(1) **Figure S1.** Structure of BI-9627 and **B)** NHE1 and uPA inhibition curves for BI-9627.

(2) **Table S1.** POLARstar OMEGA settings used for plate reader uPA and NHE1 inhibition assays.

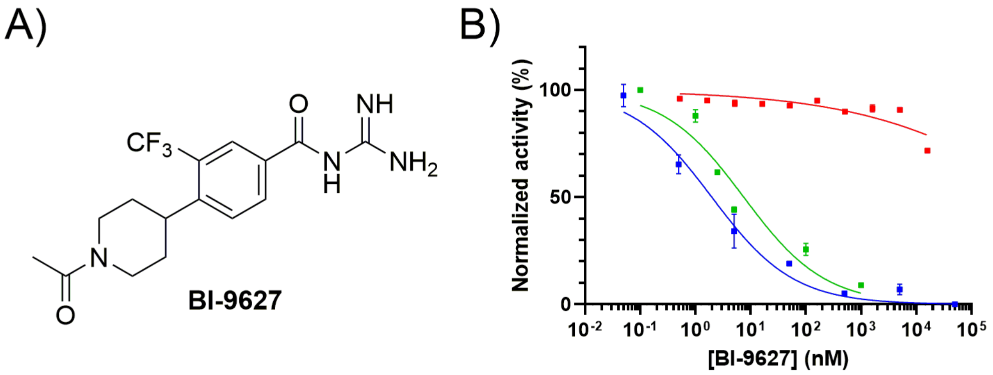

Figure S1. Structure of BI-9627 and **B)** NHE1 and uPA inhibition curves for BI-9627. Blue = NHE1 inhibition determined using the plate reader assay. Data points = mean ± SEM (n = 3 technical replicates, IC_{50} of curve shown = 1.4 nM). Green = NHE1 inhibition determined using the cuvette assay. Data points = mean ± SEM (n = 6 technical replicates/concentration, IC_{50} = 7.5 nM). Red = inhibition of human uPA activity determined using the reported fluorometric method [25]. Data points = mean ± SEM (n = 3 technical replicates). IC_{50} was not reached at the highest concentration tested (50 µM).

Table S1. POLARstar OMEGA settings used for plate reader uPA and NHE1 inhibition assays. [a]biochemical assay using low molecular weight active human uPA. [b]cell-surface assay using exogenous HMW active human uPA.

**Parameter**	**Value**		
uPA inhibition with Z-Gly-Gly-Arg-AMC			
Measurement type	fluorescence intensity, top optic		
Read mode	single excitation, single emission		
No. of cycles	60		
Cycle time	60 seconds		
Scan mode	well average		
Scan diameter/ No. of flashes	[a]whole well averaging, 20 flashes per well		
Plate shake mode	[a]double orbital, 3 s prior to each read (600 rpm), [b]off		
Assay temperature	37 ºC		
Filter settings	Excitation	Emission	Gain
	355 ± 10 nm	450 nm	900
NHE1 inhibition assay with BCECF-AM			
Measurement type	fluorescence intensity, top optic		
Read mode	plate multichromatic		
No. of cycles	5		
Cycle time	61 seconds		
Scan mode	orbital averaging		
Scan diameter/ No. of flashes	1 mm/ 8 flashes per well		
Plate shake mode	off		
Assay temperature	37 ºC		
Filter settings	Excitation	Emission	Gain
1. pH-sensitive couple	485 ± 12 nm	520 nm	1100
2. Isosbestic couple	440 ± 10 nm	520 nm	1100
